# To Be or Not to Be Solitary: *Phytophthora infestans*' Dilemma for Optimizing its Reproductive Fitness in Multiple Infections

**DOI:** 10.1371/journal.pone.0037838

**Published:** 2012-06-01

**Authors:** Julie A. J. Clément, Hélène Magalon, Isabelle Glais, Emmanuel Jacquot, Didier Andrivon

**Affiliations:** Institut of Genetic Environment and Plant Protection, Institut National de la Recherche Agronomique - Agrocampus Ouest - University of Rennes 1, Le Rheu, France; University of Liverpool, United Kingdom

## Abstract

The success of parasitic life lies in an optimal exploitation of the host to satisfy key functions directly involved in reproductive fitness. Resource availability generally decreases over time with host mortality, but also during multiple infections, where different strains of parasite share host resources. During multiple infections, the number of parasite strains and their genetic relatedness are known to influence their reproductive rates. Using infections of the potato plant *Solanum tuberosum* with the parasite *Phytophthora infestans*, we set up an experimental design to separate dose effects (double- vs. single-site infections) from genetic relatedness (different vs. identical genotypes) on the reproductive fitness of competing parasite genotypes. We showed the existence of two basic response patterns - increase or decrease in reproductive fitness in multiple infections- depending on the parasite genotype. In all cases, the intensity of the response of any genotype depended on the genotype of the competing strain. This diversity of responses to multiple infections is probably maintained by the fluctuating frequencies of multiple infections in nature, arising from variations in disease pressure over the course of an epidemic and between successive epidemics. It allows a rapid response of parasitic populations to changing environments, which are particularly intense in agricultural systems.

## Introduction

Understanding why and how infectious diseases develop and evolve is a crucial issue to improve their management. Because the main concern with infectious diseases is the disease outcome itself, most studies focus on the evolution of parasites towards greater or lesser virulence (*i.e.* pathogen-induced host damages or death), rather than on the parasite life history strategy. Virulence resulting from the expression of pathogen traits linked to host exploitation is assumed to evolve quickly, and to respond to ecological and environmental factors such as within-host multiple infections [1], [2].

Multiple infections are usually defined as the simultaneous infection of a single host by several genotypes of a parasite species [3]. Improved detection methods (particularly molecular technologies) have revealed that multiple infections are frequent in natural populations [4], [5], [6]. This finding stimulated evolutionary ecologists to consider the role of multiple infections in the evolution of parasite life history traits, and many theoretical and empirical studies now target the shift of optimal virulence during multiple infections. Classical theoretical models predict an increase of parasite virulence over time [3], [7], [8], [9], so that the faster exploiter (*i.e.* generally the most virulent strain) has a competitive advantage over the more prudent genotypes (generally the less virulent). However, empirical studies did not always support these predictions; their results indeed sometimes suggest a competitive advantage of the most virulent [10], [11], [12], [13], [14], but in other instances of the least virulent strains [13], [15] or exhibited more complex patterns suggesting interference [16], [17], [18].

Focusing on virulence evolution when dealing with the outcome of competition is fully justified, but attention should be paid to plastic parasite strategies adopted in response to multiple infections. In particular, both the reproductive strategy and fitness of the parasite have direct consequences on the epidemic dynamics. Although in most cases both strains involved in multiple infections suffer from competition (i.e. replication rates for each parasite are lower in multiple than in single infection) [10], [11], [12], [13], [16], [17], [19], the host often carries higher total parasite density [10], [17], [19], [20] in multiple than in single infections. Multiple infection can also enhance the replication rate of only one parasite genotype, leading to higher final density [15]. Within-host interactions, particularly as they affect pathogen replication strategies, are thus important to take into account for the evolution of pathogen life history traits [21] and for host-pathogen dynamics [22].

The major problem when comparing single vs. multiple infections lies on the difficulty to dissociate several nested effects: the dose effect, the number of infection sites, and the number of co-infecting parasite genotypes and their genetic relatedness. This combination of factors can lead to erroneous interpretations of the infection outcome. For example, theoretical approaches predicted that the parasite virulence level should be displaced as a function of genetic relatedness of strains involved in multiple infections [8], [23], and recent experimental studies considered the impact of parasite relatedness on parasite traits such as prevalence [24] or spore production [18]. The extreme case of relatedness between infecting strains is the infection of a host by several inoculations of a unique parasite genotype. Unfortunately, most experiments used to address issues relative to multiple infections do not actually test multiple-site infections by the same parasite genotype, but rather a multiple-dose effect [11], [19], [25] as they rely on a single-site infection with increasing inoculum concentrations. This remains an unsolvable problem due to the kind of host-parasite system used in these studies, which not allow the test of single-site vs. multiple-site infections with the same parasite genotype.

In this study, we focused on the consequences of multiple infections on the reproductive outcome of the parasite, and set to dissociate (i) the effects of single-site from double-site infection and (ii) the impact of a challenge between an identical vs. a different genotype during double-site infections, by using an appropriate plant–parasite system.. We performed artificial single-site infections (SSI) and double-site infections (DSI) using five genotypes of the oomycete *Phytophthora infestans* (Table 1), a major pathogen of potato *Solanum tuberosum* [26], and a susceptible clone (cultivar Bintje) of the host. We controlled the amount of resources by offering limited area of host foliage tissue. We hypothesized that, sharing limited resources during DSI would alter the optimal strategy of resource allocation of each genotype on its own, and consequently reproductive fitness. Although *P. infestans* is able to reproduce sexually when compatible genotype (A1 and A2 mating types) come into contact [27], we prevented sexual reproduction to occur during multiple infections by inoculating only non-compatible strains. By carrying out the experiments this way, we eliminated sex as an extraneous factor that could mask the real effects of multiple infections on asexual reproductive fitness [28].

**Table 1:** Characteristics of the five *Phytophthora infestans* genotypes used in the experimental setup.

Parasite genotype	Mating type	Sampling year	Sample origin	Virulence profile	Avr3a genotype
BEK	A1	2005	North France	1 3 4 6 7 10 11	G_241_/G_312_
BP3	A1	2005	North France	1 4	A_341_/T_312_
P13	A1	2008	West France	1 3 4 7 10 11	G_241_/G_312_
P43	A1	2008	West France	1 3 4 7 8 10 11	G_241_/G_312_
PON05	A1	2008	West France	1 3 4 7 10 11	G_241_/G_312_

All isolates were chosen for having the same mating type. The virulence profile corresponds to the ability of pathogen genotype to infect potato genotype containing resistance genes (numbered from 1 to 11). Avr3a genotype is given for the two single nucleotide polymorphisms at the positions 241 and 312 of the avr3a gene, which are responsible for the virulence profile towards the resistance gene number 3 (named R3). A_341_/T_312_ genotype corresponds to a parasite genotype unable to infect a potato genotype containing the R3 resistance gene (see virulence profile of BP3), whereas G_241_/G_312_ genotype corresponds to parasite genotype able to overcome the R3 resistance gene (see virulence profile of all but BP3).

Based on previous theoretical and empirical studies, we expected to find an increased level of competition when increasing the number of infection sites and when inoculating different instead of identical genotypes. More precisely, we expected little or no competition when comparing the reproductive outcome of SSI and DSI with a single genotype (DSI-sg), but stronger competition effects for DSI with multiple genotypes (DSI-mg) than for DSI-sg.

## Methods

### Host-pathogen system

#### *Phytophthora infestans* life cycle

*P. infestans* is a filamentous hemibiotroph pathogen, requiring living host tissue to initiate its development [26]. Infection is due to zoospores (uninucleate swimming spores), present on the host foliage and that germinate and penetrate host tissue. The pathogen establishes through an unavoidable phase of exclusive mycelium growth, during which no sporulation is possible (latent period). Mycelium growth and asexual spore production then occur simultaneously, leading to the radial expansion of the sporulating lesion until the whole host tissue is colonized. Sporulation consists in the production of sporangia, the structures allowing pathogen dissemination and containing the zoospores. The life history of *P. infestans* is thus governed by a strong constraint on resources allocation between growth and reproduction [29], [30], [31], [32]. *P. infestans* is a heterothallic species, where te simultanenous presence of both compatible mating types (named A1 and A2) leads to the development of sexual organs. Sexual reproduction leads to the formation of oospores, that are thick-walled resting organs that can survive for up to 10 years in soil [33]. The occurrence of sexual reproduction then adds an extra constraint upon resource allocation for the pathogen.

### Experimental material

Five *Phytophthora infestans* genotypes, sampled from the two major basins of potato production in France, were chosen from our lab collection (Table 1): BP3, BEK, P13, P43 and PON05. We selected them to be of the same mating type (A1), which allowed us to control the absence of sexual reproduction. We took advantage of allelic differences at the avr3a gene to easily quantify asexual spores of BP3 on one hand, and of the other four genotypes on the other hand using a quantitative PCR tool we developed [34]. Genetic differences between alleles lie in two single nucleotide polymorphisms (SNP) at the positions 241 (A to G) and 312 (T to G) of the avr3a gene (Table 1) and the genotypes we used were homozygote at these loci. We thus used BP3 as a reference genotype for all multiple infection experiments.

Because the potato is vegetatively propagated, cultivars are clones. We used the cultivar Bintje, susceptible to all isolates of *P. infestans*. Plants were grown from tubers in 12-cm-diameter pots, in a glasshouse maintained at a minimum of 18°C, under natural light supplemented with sodium lamps for a 16h-photoperiod. They were fertirrigated weekly with a 7∶12∶40 N∶P∶K fertilizer solution. For biotests, leaflets approximately similar in size were picked from fully expanded leaves from the median part of 6- to 8-week-old plants. They were transferred to the laboratory in watertight boxes to prevent drying before inoculation.

### Experimental setup

The five *P. infestans* genotypes, maintained as axenic cultures, were first multiplied separately once on potato leaves before starting the experiments. To this end, suspensions of sporangia were prepared by flooding 4-week-old cultures on pea agar dishes in 5 mL of deionised sterile water (DSW). Suspensions were kept at 4°C for approximately 3 h to promote zoospore release. Leaflets from 8-week-old plants, placed on the lids of inverted Petri dishes containing water agar to obtain near 100% relative humidity, were inoculated by depositing droplets containing about 1000 sporangia each. After 8 days of incubation under controlled conditions (18–15°C day/night temperature, 16 h daylight), newly formed sporangia were washed from leaflets in 10 mL DSW. The concentration of the resulting suspensions was adjusted to 5.10^4^ sporangia.mL^−1^ using a haemocytometer [35]. Calibrated suspensions were inoculated onto detached leaflets to generate single- and double-site infections. Droplets were always deposited onto the middle right (or left) side of the main vein of each leaflet. For DSI, droplets were deposited always at the same distance from each other. In SSI, each strain was inoculated in front of a DSW droplet. In DSI, we inoculated either two droplets of the same suspension (DSI-sg) on each side of the main vein of the leaflet, or one droplet of BP3 on one side and one droplet of one of the four other genotypes on the other side (DSI-mg). All genotypes but BP3 therefore only competed with the BP3 in DSI-mg; BP3 competed with each of the 4 other strains. This experimental setup allowed to fix the competitor identity when testing the response of different strains to multiple infections, and to test the response of one strain when competing with different genotypes. The whole experimental setup thus included 14 treatments (5 SSI+5 DSI-sg+4 DSI-mg), each repeated 12 times, for a total of 168 experimental units. Inoculated leaflets were incubated for 10 days as described for inoculum multiplication. At the end of the experiment, the whole leaflet area was colonized by sporulating lesions. Spores were then collected by washing leaflets in 10 mL DSW. A sample of 2 mL of the resulting suspension was centrifuged for 10 minutes at 13000 rpm, and the supernatant was removed. DNA extraction was performed on the pellet with the NucleoSpin 96 Tissue Core Kit (MACHEREY-NAGEL GmbH & Co. KG), and 1 µL of purified DNA was quantified with the qPCR protocol we developed [34], which allowed a suitable quantification of zoospores, the uninucleate structures from asexual reproduction.

### Defining competitiveness

We used several measures of competitive outcome for the study of individual strategies. First, relative competitiveness was calculated for each genotype in DSI-mg as the zoospore density of the target genotype divided by the total zoospore density per leaflet (sum of the target and competitor genotypes) following Bell et al. [19]. It gives the frequency of each genotype for the interactions we tested.

We also compared observed values of zoospore production of each genotype in mixed infections to theoretical values calculated assuming no competition. Let 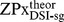 be the theoretical zoospore production of genotype X in DSI-sg, and expressed as:
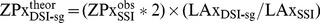
(1)where 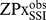 is the observed zoospore production in SSI of genotype X, and 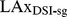 and 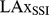 the available leaflet areas in DSI-sg or SSI respectively. Assuming no competition, the theoretical zoospore production on a double infected leaflet is expected to be doubled, hence the multiplicative factor 2 for 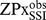. In the same way, let 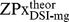 be the theoretical zoospore production in multiple genotype infection of genotype X, expressed as:
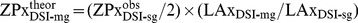
(2)where 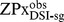 is the observed zoospore production in single genotype infections of genotype X, and 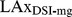 and 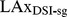 the available leaflet areas in DSI-mg and DSI-sg, respectively. 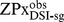 is then the total zoospore production of both inoculation points of genotype X on the leaflet. So, supposing no effect of the competitor genotype identity, the value has to be corrected by a factor 2. We chose to express 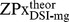 as a function of 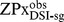 and not 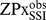 directly, because DSI-sg is the direct reference (avoiding dose effects) for a DSI-mg. We then defined two competition coefficients, κ_DSI-sg_ and κ_DSI-mg_, by comparing observed values to theoretical ones, and defined as:
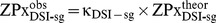
(3)

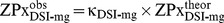
(4)These measures of competitive ability are indicators for the type of interaction. κ equals zero, when the competitor totally suppresses the reproduction of genotype X; κ lies between 0 and 1 when the competitor has a negative impact on the reproduction of genotype X; κ equals 1 if the competitor has no impact on reproduction of genotype X; and κ is greater than 1 when the reproductive performance of genotype X is enhanced in the presence of a competitor.

Combining equations [3] and [4] allow to express 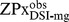 as a function of 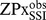 and κ_DSI-sg_×κ_DSI-mg_, where the latter is the total competition coefficient (κ_total_). This allows the decomposition of competition outcomes into one coefficient describing the effect of double-site infections (κ_DSI-sg_), and another describing the effect of genetically distinct infections (κ_DSI-mg_).

### Statistical analyses

All statistical analyses were performed using the general statistical software package RGUI version 2.11.1 [36]. Data on zoospore density or zoospore production were log-transformed to meet normality and homogeneity-of-variance assumptions for analysis of variance. Contrasts were used to test specific hypotheses in subsets of the total dataset. We always constructed full models, including all relevant variables and their interactions, and removed those that were not significant (P>0.05) to fit the most parsimonious model.

We performed two kinds of analyses based on data collected during the experiment. First, we tested the impact of different factors (number of infection sites, genetic similarity of competing genotypes, …) on the total zoospore density (zoospore number per mm^2^ of leaflet; Table 2i). These analyses were based on the total spore numbers produced per unit of plant tissue, without separating the contribution of each competing genotype.

**Table 2:** Analyses of variance contrasts for global (i) and individual (ii) reproductive strategy.

	i- Global strategy			ii- Individual Strategy			
	Total zoospores density (log-)			Total zoospores production (log-)			
	df	F	*P*	*focus on*	df	F	*P*
Infection treatment contrasts							
A- Infection mode (SSI, DSI-sg or DSI-mg)	2	33.830	**<0.001**	*BP3*	2	3.660	**<0.05**
				*Others*	2	29.496	**<0.001**
B- Number of infection sites (SSI or DSI)	1	12,308	**<0.001**	*BP3*	1	7,311	**0,009**
				*Others*	1	17,885	**<0.001**
C- Number of infection sites for a same genotype (SSI or DSI-sg)	1	0,047	0,828	*all*	1	0,097	0,757
D- Number of ompeting genotypes (DSI-sg or DSI-mg)	1	61,575	**<0.001**	*BP3*	1	0,121	0,730
				*Others*	1	42,581	**<0.001**
Pathogen Genotype contrasts							
E- Pathogen identity wihtin SSI	4	4,444	**0,004**	*all*	4	4,743	**0,003**
F- Pathogen identity within DSI-sg	4	6,502	**<0.001**	*all*	4	5,556	**<0.001**
G- Challenger identity within DSI-mg	3	15,145	**<0.001**	*BP3*	3	1,368	0,270
H- Challenger strategy within DSI-mg	1	43,076	**<0.001**	*BP3*	1	1,778	0,191
Interactions							
I- Pathogen genotype * A	4	10.057	**<0.001**	*BP3*	-	-	-
				*Others*	6	10.458	**<0.001**
J- Pathogen genotype * B	4	15,307	**<0.001**	*BP3*	-	-	-
				*Others*	3	15,662	**<0.001**
K- Pathogen genotype * C	4	9,311	**<0.001**	*all*	4	9,010	**<0.001**
L- Pathogen genotype * D	3	2,639	0,0539	*BP3*	-	-	-
				*Others*	3	2,586	0,058
M- Pathogen strategy * B	1	59,358	**<0.001**	*BP3*	-	-	-
				*Others*	1	46,626	**<0.001**
N- Pathogen strategy * C	1	34,380	**<0.001**	*all*	1	32,598	**<0.001**
O- Pathogen strategy * D	1	59,358	**<0.001**	*BP3*	-	-	-
				*Others*	1	7,550	**<0.001**

Non-significant interactions were removed from models before computing F statistics and significance of other factors. Bold typeface indicates significant effects (P-value<0.05).

Then, we tested the effect of the same factors on zoospore production by each genotype, to analyze individual strategies (Table 2ii). In this part, we made the distinction between competing genotypes thanks to the quantitative molecular method we designed. The experimental setup was designed to test simultaneously two hypotheses concerning individual strategies. On one side, we tested the stability of genotype response to multiple infections depending on the genotype of the competitor. This was done by focusing on the BP3 individual strategy during multiple infections (see focus on “BP3” in Table 2ii). On the other side, we tested the diversity of responses among different genotypes faced to a single, reference competitor (here BP3). This was done by testing individual strategies of the other four genotypes during multiple infections (see focus on “Others” in Table 2ii). In each statistical analysis, only required data were used to design the statistical models (for example we used only BP3 data when focusing on the stability of genotype response). Results shown in Table 2 were always for the most parsimonious model. All P-values below 0.05 (in bold in Table 2) indicated significant differences in zoospore production between levels of the factor tested.

We also tested whether the relative contribution of BP3 to the total asexual reproduction differed depending on the challenger, by fitting a general linear model with a quasi-binomial error distribution. With this error distribution, the significance of the explanatory variable (challenger identity) was tested with a F test rather than a χ^2^ [37] by adding terms sequentially in the analysis of deviance.

## Results

### Infection performance

Six of the 168 inoculated leaflets failed to produce a sporulating lesion, and were thus excluded from analyses. Because our molecular test had a detection threshold of 10^2^ DNA copies.µL^−1^ [34], quantification below this value were considered as missing data. Quantification of genotype P13 offspring failed for two leaflets in SSI. Quantification of genotype BP3 offspring failed for 8 leaflets in DSI-mg, certainly due to its lower reproductive fitness compared to all the challengers.

### Total reproductive fitness

**Figure 1:**
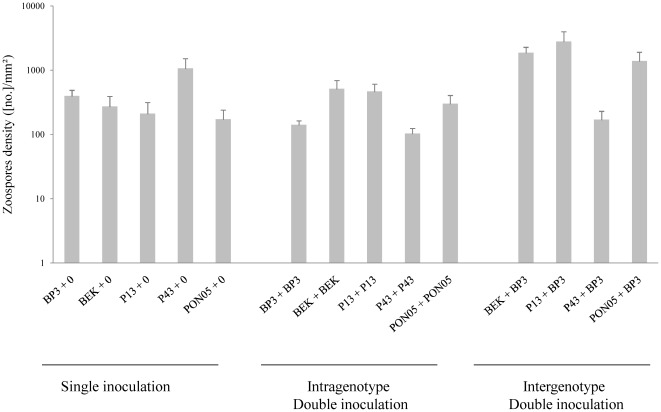
Overall zoospore density (Means+SE) in single-site infection (SSI), double-site infection of single genotype (DSI-sg) and double-site infection of multiple genotypes (DSI-mg).

Infection treatments (SSI, DSI-sg or DSI-mg) affected the overall zoospore density of *P. infestans* (Table 2i A). Inoculation with two genotypes instead of one (DSI vs. SSI) always significantly altered total zoospore density (Table 2i B, Figure 1), but the direction of change depended on the genotypes (or combination of genotypes), as revealed by the significant interaction term (Table 2i J). No significant overall difference between SSI and DSI-sg was demonstrated (Table 2i C), due to different responses between genotypes, as shown by the interaction term (Table 2i K). In fact, two genotypes (BP3 and P43) produced fewer zoospores in DSI-sg than in SSI, while the other three (BEK, P13 and PON05) produced more zoospores in DSI-sg than in SSI (Figure 1). We designated the first group of genotypes as ‘solitary’ and the second group as ‘solidary’, based on their response to DSI-sg. This new factor (solitary or solidary response to DSI) explained the quantitative variation in total reproductive fitness between SSI and DSI, respectively (Table 2i M & N; Figure 1). In all cases, competition between genetically different parasites enhanced the total reproductive fitness compared to the challenge between identical genotypes (Table 2i D, L & O; Figure 1), without changing the direction of change (increase or decrease) compared to SSI. The total reproductive fitness in DSI-mg clearly depended on the challenger genotype (Table 2i G), and particularly on their reproductive strategy (*i.e.* solitary or solidary; Table 2i H).

### Individual reproductive strategies

**Figure 2:**
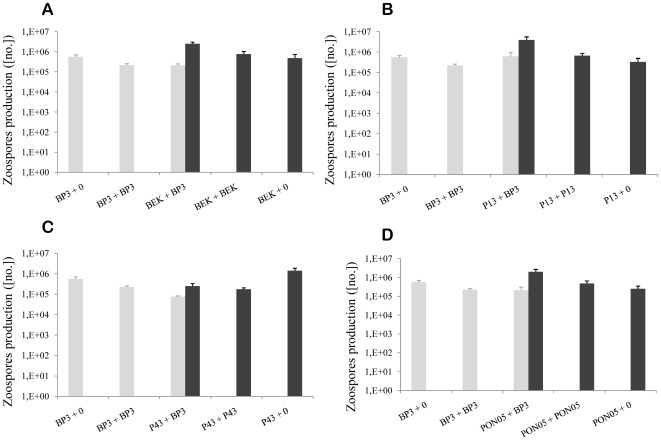
Individual zoospore production per leaflet (Means+SE) in single and multiple infections for the BP3 reference genotype (grey bars) and BEK (A), P13 (B), P43 (C) and PON05 (D) genotypes (black bars).

Significant differences between individual reproductive fitness in SSI and DSI-sg (and between SSI and both DSI conditions) indicated that overall differences described above were due to individual fitness changes (Table 2ii A, B & C; Figure 2). The nature of these differences was clearly dependent on the genotype (Table 2ii I, J & K; Figure 2), and even more on the strategy (solitary or solidary) of the genotype (Table 2ii M & N).

**Figure 3:**
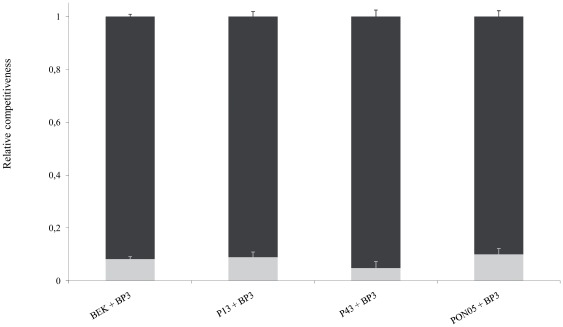
Relative proportion (Means+SE) of zoospores density of BP3 (grey bars) and challenger (black bars) genotypes in double-site infection of multiple genotypes (DSI-mg).

The reproductive fitness of BP3 was not affected by the genotype of the challenger inoculated during DSI: no significant differences were found between DSI-sg and DSI-mg involving BP3 (Table 2ii D), between different challenger genotypes (Table 2ii G) or between challenger strategy (Table 2ii H). Moreover, in all DSI-mg, the relative proportion of BP3 asexual offspring did not differ according to either the challenger genotype (F_3,33_ = 1.91, P = 0.15) or their strategy (F_1,35_ = 0.08, P = 0.78), and reached 8.24% in average (Figure 3).

The reproductive fitness of the other four genotypes during DSI depended on the genotype of the second genotype inoculated (the genotype itself or the reference challenger BP3; Table 2ii D, Figure 2). The genotype strategy clearly played a role in the reproductive outcome (Table 2ii O): the three solidary genotypes were fitter when challenged with BP3 than in self-competition, whereas the solitary P43 genotype was not (Figure 2).

**Figure 4:**
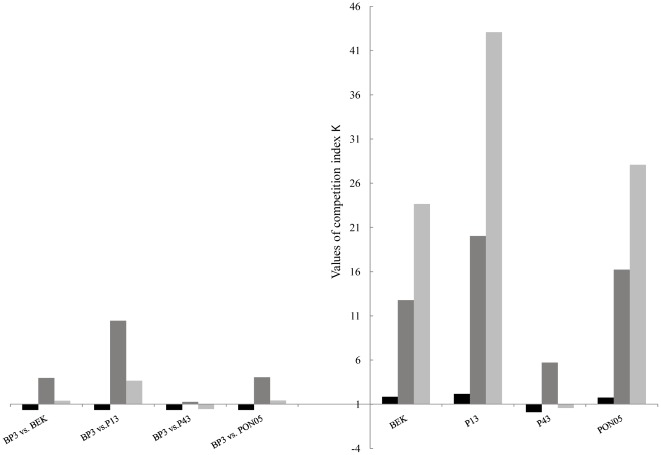
Competitive coefficients assessing the deviation of observed zoospore production (ZP_obs_) to theoretical value (ZP_theor_) supposing no competition. κ_intra_ (black bars) measured the deviation of ZP_obs_ in double site infection with a single genotype (DSI-sg) to ZP_theor_ estimated from ZP_obs_ in single site infection (SSI). κ_inter_ (dark grey bars) measured the deviation of ZPobs in double-site infection of multiple genotypes (DSI-mg) to ZP_theor_ estimated from ZP_obs_ in DSI-sg. κ_total_ (light grey bars) combined both κ_intra_ and κ_inter_. κ>1 indicated that zoopore production is enhanced, 0<κ<1 indicated that zoospore production is reduced (*i.e.* there is competition) and κ = 0 indicated that zoospore production is totally suppression.

Values of the κ_DSI-sg_ competition index depended on the response pattern of the genotypes. This index was below 1 for the two solitary genotypes BP3 and P43, but ranged between 1.73 and 2.15 for the solidary genotypes BEK, P13 and PON05 (Figure 4). In all interactions and for all genotypes, challenging a different genotype always resulted in an increase of the reproductive fitness compared with challenging itself (κ_DSI-mg_>1, Figure 4). Moreover, the intensity of this increase depended on the genotype strategy (κ_DSI-mg_<11 for solitary and κ_DSI-mg_>11 for solidary) and on the BP3 challenger strategy (κ_DSI-mg_>4 when the challenger is a solidary genotype, κ_DSI-mg_ = 1.28 for BP3 challenged with the P43 solitary genotype; Figure 4). The κ_total_ composite index illustrated the variation in the reproductive fitness during DSI-mg compared to SSI. Thus, even if the challenge with a genetically distinct parasite always resulted in an increase in asexual reproduction, the interaction outcome was clearly linked to the genotype strategy and that of its challenger (Figure 4).

## Discussion

Reproductive strategies of plant pathogens in response to DSI-sg and DSI-mg have to our knowledge rarely been studied. In our experiment, we tried to disentangle the multi-site infection effect from the impact of sharing host resources with another genotype (i.e. multiple infections *sensu stricto*). The particular life cycle of the filamentous spore-producing pathogen we used was well suited for this. Indeed, unlike other parasites that have been abundantly studied in multiple infections, *P. infestans* is not a within-host free living organism. After inoculation, it has to penetrate host tissue and to invest in mycelial growth to move within the host and to acquire the nutrient resources it needs for reproduction. This specificity allowed to dissociate a double dose inoculation from a double-site infection (DSI) with the same genotype (DSI-sg), and thus to really test double genotype infections (DSI-mg).

As predicted from theoretical studies, we found that DSI could lead to a decrease in reproductive fitness, but only for some of the genotypes we tested. In fact, we surprisingly highlighted that other genotypes of *P. infestans* responded differently to multiple infections by enhancing their reproductive investment. Our results thus showed the diversity of responses to multiple infections among *P. infestans* genotypes. More specifically, we found either higher or lower overall asexual zoospores density for DSI compared to SSI, depending on pathogen genotypes and their specific combination. Either higher [7], [13], [15], [16] or lower [8], [12] level of total parasite density within the host have been demonstrated experimentally for different systems. If asymmetric competition was observed between closed species of nematodes in the genus *Steinernema* [14], to our knowledge, it had never been shown that both competition outcomes could occur within a single species. We also had the ability to assess individual reproductive fitness in DSI-mg (through a genotype specific quantitative PCR tool) and thus to determine the relative contribution of each genotype to total offspring density. These results confirmed that individual investment in asexual reproduction depended on the infection mode, the pathogen genotype and the challenger genotype. They also revealed different patterns of genotype response to multiple infections.

Two clearly different strategies to multiple infections were highlighted in this experimental study. Solidary genotypes (*i.e.* BEK, P13 & PON05) displayed up to 40 times higher reproductive fitness in multiple than in single infection, while solitary genotypes (*i.e.* BP3 & P43) reproduced significantly better when inoculated alone (Figure 3 & 4). Moreover, solitary genotypes were fitter in SSI compared to solidary genotypes (Figure 2).

The total infection outcome in DSI clearly depended on the strategy of both competing genotypes. These conclusions apply to both DSI-sg and DSI-mg. This is why the combination of two solitary genotypes (*e.g.* BP3+P43 or BP3+BP3) decreased the overall but also individual reproductive fitness (Figure 2 & 3C), while the interaction of two solidary genotypes (e.g. P13+P13, Figure 3B & 4) enhanced the reproductive fitness. Interestingly, the interaction between genotypes with opposite strategies seemed to stimulate the “solitary” genotype, resulting in a higher (although not statistically significant) asexual reproduction (see *e.g.* BP3 faced to P13, Figures 2B & 4).

In all multiple infections, each interacting partner reproduced better when confronted with a different genotype than when confronted with itself. This means that *P. infestans* is able to recognize itself, and to adopt a different reproductive strategy in response to this detection. We know that recognition between compatible strains is possible through hormonal exchanges [29], but mechanisms for distinguishing self vs. non-self within a mating type are not known. This conclusion could never have been reached without decomposing DSI-sg from DSI-mg.

### Putative mechanisms of variation in asexual reproduction investment

Asexual reproduction in *P. infestans* is known to be plastic in single infections. Different constraints lie behind this plastic response, including abiotic [38] and biotic [35] factors. Our data support the idea that the number of infection foci greatly influences the investment in asexual reproduction. How the pathogen adapts its response to the imposed sharing of resources remains unclear, but several hypotheses exist [21]. First, the pathogen could enhance host exploitation either by diversifying its resource uptake from the host or by improving its ability to acquire these resources. Second, modifying the resource allocation could favour asexual reproduction over mycelial growth (*i.e.* host colonization) in competition. Indeed, as for other spore-producing pathogens [39], *P. infestans* has to trade-off the resources invested in different biological functions such as growth and reproduction [29], [30], [31], [32]. Higher reproductive fitness could result from the displacement of the growth-reproduction balance, as shown in *Plasmodium chabaudi* [17]. A third way to change the reproductive fitness could be to acquire host resources as quickly as possible. Adaptation for the timing of first reproduction (*i.e.* the latent period) could impact the cumulative production of asexual spores. This had been theoretically demonstrated for such pathogens in single infection [39] but there is no prediction regarding multiple infections. This therefore remains an interesting direction for future investigations. Most life history traits take a range of values from a minimum to a maximum threshold (*e.g.* latency period in *P. infestans* cannot equal zero), and are under trade-off constraints [29], [32]; it can thus be sometimes difficult to shift towards a higher (or lower) value to adapt to multiple infections. It was evidently the case for solitary genotypes, which failed to enhance their asexual reproduction in multiple infections.

### Coexistence of strategies over time: why?

The existence and persistence of the two strategies within natural populations of *P. infestans* are probably linked to epidemic dynamics in time and space. At the beginning of epidemics, competitive pressure is low. This would favour the solitary genotypes, which have a higher fitness in single infections. However, the frequency of multiple infections likely increases over the course of each epidemic, giving the advantage to solidary genotypes, which are then fittest. Tracking the relative proportion of each strategy over the course of an epidemic could validate this scenario of geographical coexistence. If it is confirmed, it may be difficult to determine experimentally if balancing selection is indeed responsible for the existence and maintenance of these two strategies, but nested models could provide elements of response by linking within- and between-host dynamics [40].

Competitive pressure is crucially linked to population dynamics. All factors influencing population dynamics would then also impact competitive pressure. These factors are extremely diverse in agrosystems; they include climatic variables, such as temperature or humidity, as well as constraints specific to agricultural management, for example pesticides, host density or host genotype. The coexistence of several reproductive strategies possibly allows *P. infestans* to respond to rapid changes in this highly unstable environment.

### Consequences on virulence

Although virulence is assumed to be a direct consequence of within-host pathogen multiplication, this direct causative relationship can be questioned [41]. At the scale of the lesion for instance, lesion area can be regarded as one of the possible measures of virulence [42]. At the end of our experiment, every leaflet was covered by a sporulating lesion. If lesion area can be regarded as a valid proxy for virulence, multiple infections led to higher asexual reproduction rates for similar virulence level (*i.e.* a similar sporulating lesion area). This could indicate that within-host replication does not necessary correlate with a higher virulence for such pathogens. However, at the scale of one infection generation and without information about between host dynamics, it is impossible to predict the long term evolution of virulence [43].

### Conclusion

This study showed the diversity of *P. infestans* responses to multiple infections. Enhancing asexual reproduction when the partner is genetically different could thus be a form of competitive adaptation, because asexual reproduction fits with the dispersal function, but this assumption had to be confirmed. The asexual reproduction is known to be plastic regarding environmental changes. Here we demonstrated that the plasticity of this trait could be extended in response to multiple infections. As *P. infestans* is a heterothallic facultative-sexual species, multiple infections involving sexually compatible genotypes could expand our understanding of within-host dynamics and more particularly, the life history strategy of this plant pathogen.
